# DNA-Based Chemical Unclonable Functions for Cryptographic Anticounterfeit Tagging of Pharmaceuticals

**DOI:** 10.1021/acsnano.4c10870

**Published:** 2024-10-22

**Authors:** Anne M. Luescher, Wendelin J. Stark, Robert N. Grass

**Affiliations:** Institute of Chemical and Bioengineering, ETH Zurich, Vladimir-Prelog-Weg 1, Zurich 8093, Switzerland

**Keywords:** anticounterfeit, DNA, physical unclonable functions, DNA computation, authentication, cryptography

## Abstract

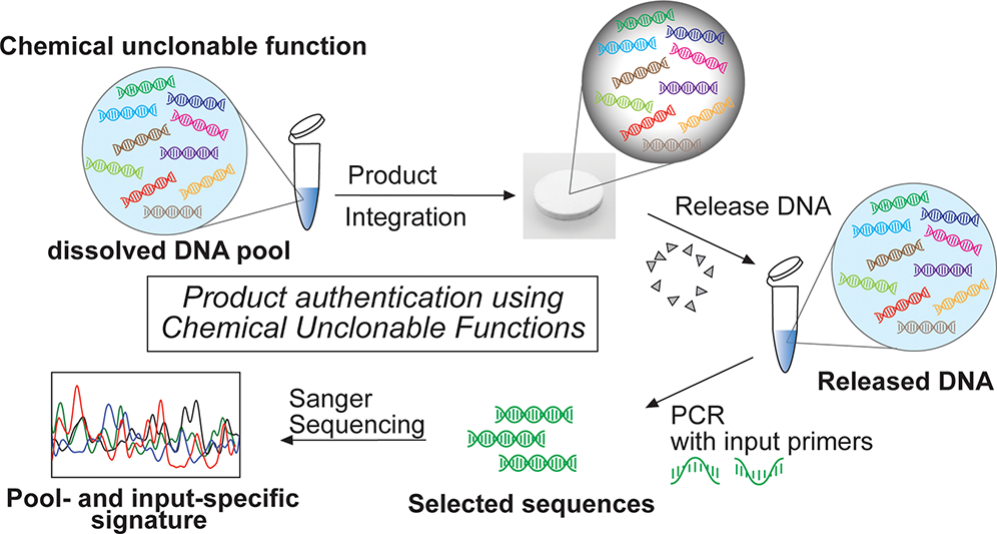

Counterfeit products are a problem known across many industries. Chemical products such as pharmaceuticals belong to the most targeted markets, with harmful consequences for consumer health and safety. However, many of the currently used anticounterfeit measures are associated with the packaging, with the readout method and level of security varying between different solutions. Identifiers that can be directly and safely mixed into the product to securely authenticate a batch would be desirable. For this purpose, we propose the use of chemical unclonable functions based on pools of short random DNA oligos, which allow the integration of a cryptographic authentication system into chemical products. We demonstrate and characterize a simplified workflow for readout, showing that results are robust and clearly differentiate between the correct tag and a counterfeit. As a proof of concept, we demonstrate the labeling of an acetaminophen formulation with a chemical unclonable function. The acetaminophen was successfully authenticated from a subsample of the product at a DNA admixing concentration of below 50 ng/g. Stability tests revealed that the readout is stable at room temperature for several years, exceeding the shelf life of most drug products. Our work thus shows that chemical unclonable functions are a valid alternative to state-of-the-art anticounterfeit methods, enabling a secure authentication scheme that is physically linked to the product and safe for consumption. The method is widely applicable beyond pharmaceuticals, allowing for more secure product tracing across industries.

## Introduction

Counterfeit products are a major consumer hazard due to the lack of quality assurance and legal accountability.^1^ This especially applies to chemical products intended for direct application on or in the body. Cosmetics and pharmaceuticals both rate among the top ten industries affected by counterfeit products,^2^ and in a WHO study, 10.5% of analyzed pharmaceuticals in low and middle income countries were substandard or falsified.^3^

Counterfeit medicines can have severe consequences for consumers, leading to illness or even death,^4^ and elaborate counterfeits can look deceptively authentic.^5^ Anticounterfeit measures are therefore widely employed, some of which are listed in Table 1 and discussed in more detail in Note S1. Importantly, these measures do not only enable authentication, but also render the production of a convincing counterfeit more costly,^6^ reducing the incentive of profitability. Examples of such measures include tamper-evident or tamper-resistant seals and product authentication features on the packaging, e.g., QR codes, RFID or specialty inks.^7^ These methods are usually applied to the primary or secondary product container, e.g., as prints or inlays, and can have a double function in anticounterfeiting and traceability.^8^ However, packaging-associated identifiers are separate from the product itself, which can be exploited by counterfeiters. It is therefore desirable to use on-dose authentication technologies instead of or in conjunction with packaging-based security.^9^

**Table 1:** Comparison of Different Pharmaceutical Identifiers and Anticounterfeit Methods

Technology	Readout	Time of verification	Information content/encoding capacity	Replicability	Level of information security	Product association
RFID^7^	Electro-magneticscan	Seconds	High (kilobits)^7,31^	Difficult^7^	High (can be encrypted, but vulnerable to attacks)^32^	Chip in/on packaging
Nanomaterials^12,13^	Magnetic, UV–vis, IR/luminescence	Seconds	Low to high (bytes to kilobytes)	Difficult^12^	Low (no encryption)	Ink print on packaging or capsule
Edible PUF^33^	Optical scan	Seconds	Extremely high (2^120^ bit)	Unclonable	Very high (random physical process)	Film printing
DNA barcode,^27,28,34^	PCR and/or sequencing	Hours	Very Low (bytes)	Moderate (effort to sequence and synthesize)	Low (no encryption)	Ink print label, product integration
DNA unclonable function (this work)	PCR, sequencing	Hours	Very high (gigabyte)	Unclonable	Very high (random physical process)	Product integration

Taggants employing encoded polymers,^10^ nanomaterials^11−13^ and microstructures^14^ have been suggested for this. Along similar lines, DNA has been widely researched as an information carrier and tag, as it has several advantageous properties. These primary benefits include its invisibility, high information density and the possibility of product integration.^15−17^ For example, various formulations of short DNA sequences have been used for labeling liquid consumer products, such as oil and milk,^18,19^ and for supply chain tracing.^20^ The exponential signal amplification through polymerase chain reaction (PCR) means the DNA tags can be detected at very low concentrations at high specificity.^21^ In addition, several technologies are available to increase the stability of DNA and make the tags more robust and durable in terms of exposure to temperature changes, radical oxygen species and UV irradiation.^22−24^ Consequently, such formulations can be invisibly, stably and homogeneously embedded into materials and objects, even if they are undergoing harsh manufacturing processes.^25^

Product-integrated DNA-based systems have also been specifically suggested for anticounterfeiting of various goods, such as currency.^26^ In pharmaceutical products, short DNA sequences have been directly integrated into lactose pills, which could then be successfully authenticated by identifying the tag.^27^ In another example, DNA markers were applied to pharmaceutical grade printer ink and then sprayed onto acetaminophen capsules as a physical chemical identifier and internalized barcode.^28^

While such short DNA strands provide product-integrated information usable for identification and tracing, the security they provide is very limited, as there is no intrinsic encryption. This is something DNA tags have in common with most other labels that are deterministically produced.^29^ Once a given product label has been identified, reproducing it is no longer a big technological or financial obstacle. Consequently, the key, or even the labeling method itself, needs to remain secret, which essentially limits the capacity of authentication to the license holder and official authorities. This also applies to steganographic approaches that additionally obscure the relevant information in a noisy background.^30^

These weaknesses can be avoided by using physical unclonable functions (PUFs). PUFs are unclonable items which, when provided with a given external stimulus (the challenge), generate an output (the response). This challenge-response relationship is rooted in truly random components of the item and therefore the result is unpredictable, but reproducible.^35^ Many PUF systems have been characterized,^36^ but oftentimes they do not qualify for pharmaceutical product integration. Edible PUFs have been developed to address this and are based on fluorescent silk microparticles that can be applied to pills via film coating.^33^ However, this approach means every dose corresponds to a separate PUF, each of which needs to be registered separately for authentication. In addition, film printing is not suitable for nonsolid delivery forms.

A technology that has the potential to close this gap are DNA-based chemical unclonable functions (CUFs), which bring together the combined advantages of distributability and unclonability.^37^ In contrast to PUFs consisting of a unique token that cannot be copied by design, CUFs can be generated in a near-unlimited number of identical copies, before being switched into an uncopiable state through biochemical processing. In theory, this enables homogeneous labeling of an entire batch, with each dose or subsample containing one or more copies of the CUF. This approach would greatly simplify manufacturing and authentication as opposed to single dose labeling with a PUF, while still providing the same anticounterfeit protection. However, DNA-based CUFs currently involve lengthy and complex readout and data processing steps, needing four PCR reactions and a next-generation sequencing (NGS) step per evaluation. In addition, their amenability for product integration and readout stability over time have so far been unknown.

Here, we show that DNA-based chemical unclonable functions can be integrated into a more low-tech workflow that is fast and cost-effective. We introduce an authentication algorithm for distinguishing different keys and unambiguously identifying a fake pool. Further, we experimentally demonstrate that silica encapsulation enables the use of CUFs for tagging and authenticating acetaminophen, which was already employed as a test substance in a previous study for DNA labeling,^28^ and is one of the most commonly used drug substances in the world.^38^ Finally, we investigate the shelf life and readout stability of the product tag over time, showing high durability. Overall, we demonstrate that CUFs can be integrated into a seamless workflow for pharmaceutical product labeling and authentication, successfully employing Sanger sequencing for simple readout and silica encapsulation for prolonged shelf life.

## Results

### Development of Authentication Scheme

**Figure 1:**
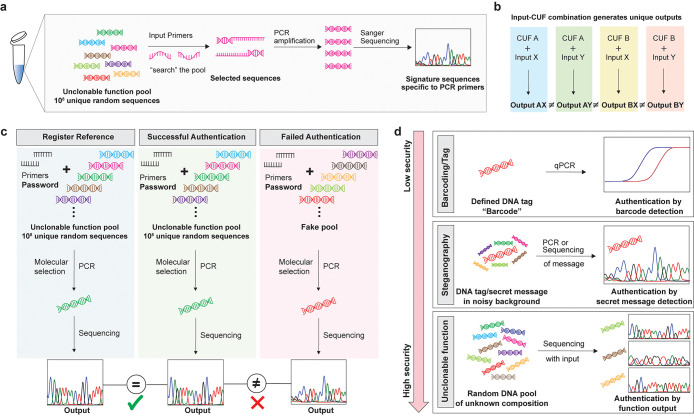
Overview of the CUF authentication system. (a) General working principle of chemical unclonable functions (CUFs). A CUF consists of a large pool of operable random DNA sequences, which are mainly structured in two input segments, two adapter segments and an output segment. To operate the function, a pair of PCR primers, the “inputs”, are added to the pool. These primers will bind to the input segments of only those sequences that perfectly match their complementary base composition. Only the selected sequences will be exponentially amplified by PCR and can then be sequenced. The Sanger electropherogram works like a signature or fingerprint for the respective input. (b) Conceptual schematic of specific output generation through combining different CUFs with different inputs. (c) Proposed authentication scheme, consisting of registering a reference sample, to which test samples are compared. A product containing the same function pool as the reference is expected to produce a highly similar readout, while a different pool generates a distinct output signal. (d) Comparison of different available DNA tagging methods, ranked from low to high security level.

DNA-based CUFs rely on random DNA pools of enormous diversity. The working principle has been previously described^37^ and is largely based on the fact that the pools are truly random in their composition^39^ and too large for the entirety of the information to be accessible. As shown in Figure 1a, the unclonable function accepts a set of PCR primers as an input, and through PCR outputs a set of random sequences from the pool. Each sequence in the pool is by design partitioned into random and constant segments (see Figure S1). Two random segments are bound by the input primers, and a third is subsequently read as the output. The constant segments facilitate further processing and sequencing. The primers thus perform a molecular selection, resulting in only a few sequences being selected and amplified out of a pool of billions, that can then be identified by sequencing. These amplified sequences are of random origin but specific to the input, i.e., the same input will always produce the same output, and different inputs will result in different outputs. Due to the randomness and vastness of the pool, it is nearly impossible to correctly predict a given output to an input, and vice versa. Crucially, an output is always the function of the specific combination between a CUF and a pair of input primers. Consequently, different CUFs produce unrelated outputs, even if the same input is used (Figure 1b). Moreover, the output to a new input-CUF combination is unknowable to anyone, including the issuer of the CUF.

The authentication scheme we propose in this work (Figure 1c) is based on first creating a positive reference by registering challenge-response pairs of the authentic CUF as added to the product. Later on, the authenticity of a sample can be assessed by performing the same challenge-response procedure. If the challenge-response-pairs match between the sample and the reference, the product can be considered authentic, while an inauthentic product will fail to produce the same response, if any output can be generated at all. In contrast to DNA barcodes or steganography, the proposed scheme derives a higher level of security from the unclonability of the DNA pool (Figure 1d).

### Evaluation and Characterization of CUFs Using Electropherogram Correlation

To implement a workflow for product authentication, we first generated a random DNA pool according to our previous work,^37^ encompassing *approx.* 10^8^ unique random sequences (for the library design and processing refer to Figure S1). Using this pool, we then developed a simplified method to measure and compare challenge-response-pairs based on Sanger sequencing electropherogram comparisons. The CUFs described in previous literature depended on a multistage process, including several library preparation steps for next-generation sequencing, followed by complex data processing.^37^ Here, we aimed to develop a simplified process by employing Sanger sequencing and an simplified evaluation method, which reduces the time and complexity of the workflow and facilitates outsourcing to a sequencing provider. These are advantages in decentralized authentication scenarios, where an individual or institution wants to securely confirm authenticity and batch information on a time scale of hours without needing extensive know-how and infrastructure. To incorporate facilitated analysis into our workflow, we developed an algorithm for electropherogram comparison to optimize data alignment and then calculate the correlation coefficient to evaluate authenticity. The method, which we validated experimentally, uses alignment of the four electropherogram traces based on identifying the constant peaks at either end of the output signal. To compensate for the variations in signal acquisition between individual experiments, the *y*-axis is then normalized, and the traces are resampled to a constant length. Only with this processing of the Sanger electropherograms, reliable correlation metrics could be established (see Figure S2). As the defining similarity metric, we use the average of the individual correlation coefficients of the four traces (for A, C, G and T).

**Figure 2:**
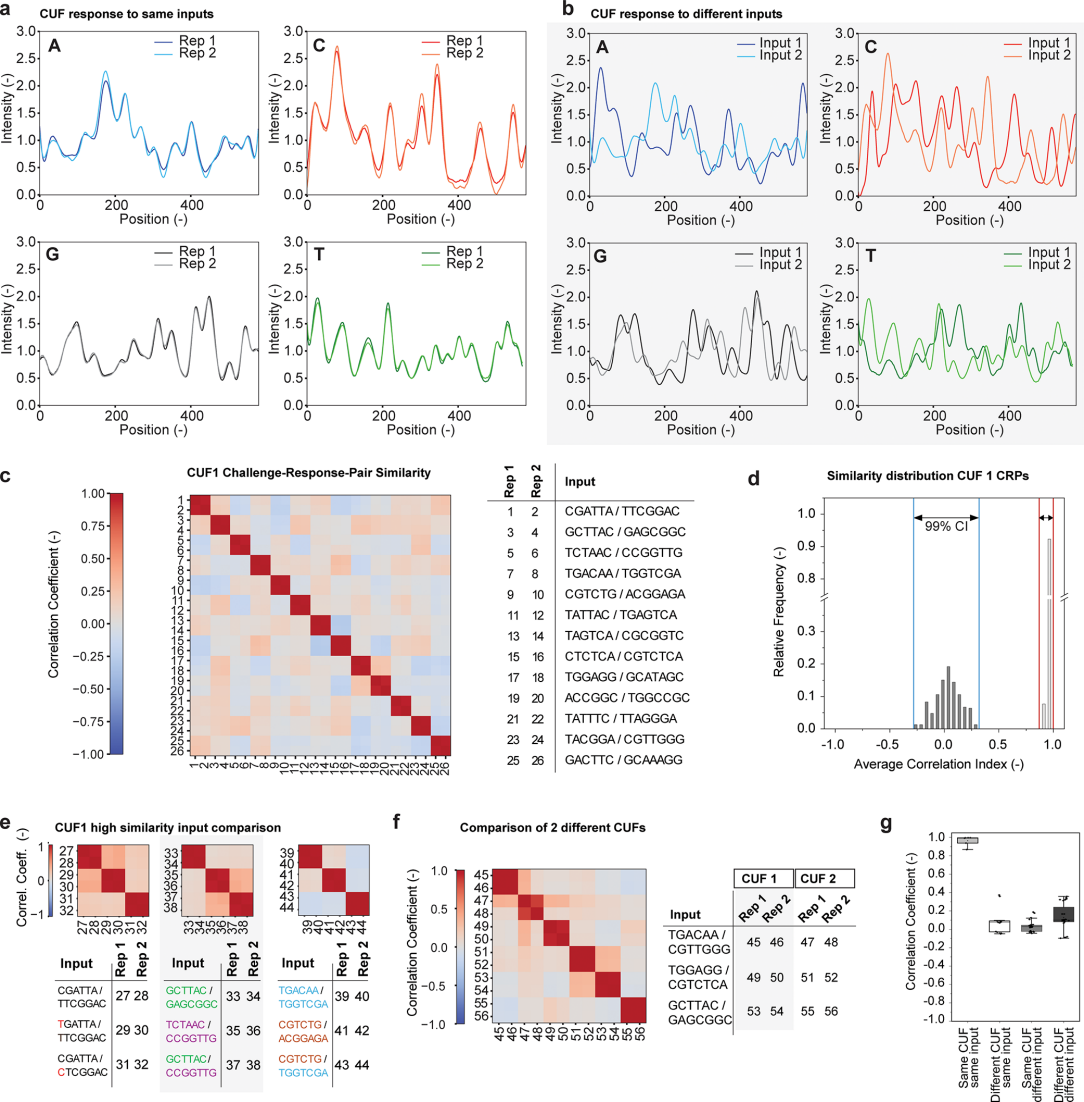
Characterization and performance analysis of chemical unclonable functions with electropherogram readout. (a) Overlay of two readouts of a chemical unclonable function with the same input after processing. The electropherograms corresponding to the four DNA bases A, C, G and T are compared between two separate executions/repetitions, demonstrating the high readout similarity. (b) Overlay of two readouts of a chemical unclonable function in analogy to (a) but using unlike sets of inputs. (c) Matrix comparing electropherogram similarity, quantified as the average correlation coefficient from all four channels. The table indicates the 13 inputs tested, with two runs each, mimicking the recording of a reference and a sample. (d) Histogram of all comparisons between readouts stemming from like inputs (intracomparisons, *n* = 13) and unlike inputs (intercomparisons, *n* = 312), respectively. The blue lines indicate the 99% confidence interval for the distribution of comparisons between different inputs, the red lines indicate the same for the distribution of identical inputs. The calculations are based on the probability density distribution of the correlation coefficient assuming a bivariate normal distribution for the samples. (e) Matrices comparing inputs that are not identical, but highly similar, as indicated by the complementary tables. Rep 1 and Rep 2 indicate the two separate reactions and measurements using the same input. (f) Matrix comparing correlation between outputs generated by two different CUF pools in response to the same inputs. The table assigns each experiment to the respective CUF and the function input, with Rep 1 and Rep 2 indicating two independent executions. (g) Boxplot showing the distributions of correlation between responses of the same CUF and different CUFs to like and unlike inputs, respectively. The data compares the outputs to 3 inputs as measured in duplicates for two different CUFs. When cross-comparing them and categorizing them into same CUF/same input, different CUF/same input, same CUF/different input and different CUF/different input, the number of comparisons amounts to *n* = 6, 12, 24, and 24 for the four boxes, respectively. Indicated are the median (horizontal line), mean (circled dot), 25th and 75th percentile (box) and 1.5 interquartile range (whiskers), with all samples individually shown as black dots.

Comparison of two independent measurements of a CUF with the same input and processing the data as described above results in matching electropherograms (Figure 2a), while the data generated from two different inputs are optically clearly distinct (Figure 2b).

To assess the performance of the function and data processing more quantitatively, we generated challenge response pairs (CRPs) using 13 arbitrarily selected input primer pairs (sequences infw1–13 and inrv1–13, as provided in Table S2). Each input was measured in two separate experiments, simulating the process of registration and authentication and resulting in a total of 26 CRPs. The resulting Sanger electropherograms of the four channels (corresponding to the four bases A, C, G and T) were aligned and cross-compared in terms of their correlation coefficient as described above. This led to a total of 325 unique cross-comparisons between experiments, shown in Figure 2c,d. The data shows that the distribution of outputs measured from different inputs is centered around the correlation of 0 (i.e., they are on average randomly correlated), while the outputs to the same input have a correlation close to 1 (i.e., they read as nearly identical).

In addition, inputs that were deliberately chosen to be close to each other were compared. Specifically, high-similarity input primers differing by the minimal alteration of 1 base, and inputs differing in only one of the two primers were tested and compared. CRPs generated from such similar inputs are expected to be particularly difficult to distinguish, as the sequence sets emerging from the PCR step are expected to have some similarity due to off-target amplification. Nevertheless, the experimental data shows that high-similarity inputs still produce clearly distinguishable outputs (Figure 2e). This suggests that the electropherogram-based evaluation is not inferior in resolution than the previously reported NGS-based approach. A single-base resolution in terms of input sensitivity means that, at the implemented scale of 10^8^ sequences and 13 input bases, 4^13^ ≈ 67 million unique CRPs can be evaluated.

### Distinguishing Different CUFs

Overall, these data show the robustness of the function in combination with the readout method and the high performance of the data processing algorithm. While robustness within a given function or tag is a prerequisite for anticounterfeiting applications, it is also paramount that another pool of equal design produces a different set of CRPs. The correct distinction between pools is a necessary requirement for distinguishing separate CUFs used to label different products or batches, as well as to identify potential counterfeits. Therefore, a second randomly manufactured CUF was subjected to 3 challenges. The responses were compared to the CRPs of the original CUF with the same inputs (Figure 2f), showing that the same inputs produce different outputs in the two pools. The correlation among different CUFs run with the same input is within the range of the variations observed between different CRPs within the same pool and those between different CRPs measured with different pools (Figure 2g). This is despite the fact that these experiments are highly contamination-sensitive, and all tests were conducted on the same equipment without spatial separation and with only a minimal time difference. Consequently, different CUFs reliably produce different outputs when exposed to the same inputs, meaning a single randomly chosen CRP is sufficient to distinguish two given CUFs from each other.

### Determination of the Minimal Copy Number for Successful Readout

For product labeling purposes, it is advantageous to know the minimal amount of taggant needed for successful readout. As the readout is a function of the multitude of sequences in the pool (with the primers selecting a subset, which generates a cumulative signal), the majority of sequences need to be present in the analyzed sample. If one or several of the sequences strongly contributing to the signal are not accessible, the electropherogram will become distinct from the reference, potentially leading to a false negative result. This is a stochastic process, as the frequency of individual sequences in a picked sample is expected to be Poisson-distributed and will additionally be subject to PCR bias. While this cannot be quantified exactly due to the random and unknown pool composition, it means that more than an average copy number of 1 (i.e., 10^8^ sequences in the reaction) is required to cover all input-output combinations. At lower copy numbers the signal specificity is expected to decline.

**Figure 3:**
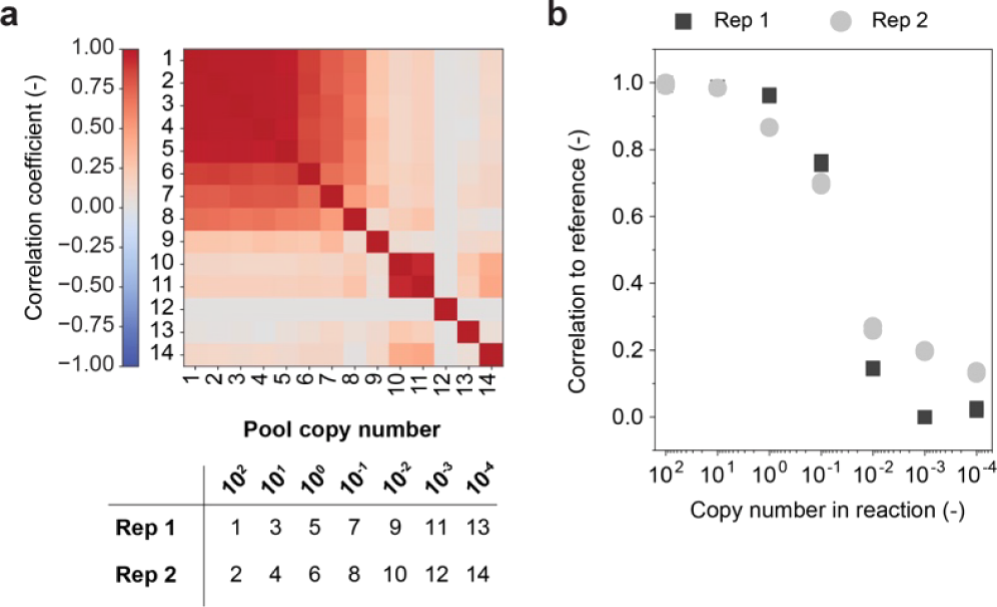
Response similarity in dependence of copy number. (a) Matrix comparing outputs measured with a constant input at different DNA concentrations, with the pool copy number ranging from 10^2^ to 10^–4^. The matrix shows correlation coefficients calculated as the mean from all four bases, with the table indicating which experiment belongs to which dilution. Two repetitions (Rep 1, Rep 2) per concentration were performed. Sample 12 did not generate a sufficient PCR signal and was set to a default similarity of zero. (b) Correlation of outputs as produced from different copy numbers of the same CUF. The correlation of each copy number to the reference is shown, whereby the reference is the output measured with the highest copy number.

This was experimentally confirmed by measuring a dilution series, ranging from an average of *approx.* 100 copies (the same amount used for previous CUF characterization experiments, as estimated from DNA concentration measurements) down to only 10^–4^ copies. All dilutions were subjected to PCR with the same input primer pair, followed by comparison of the correlation coefficients with the highest concentration serving as an internal positive reference. Figure 3a shows the correlation matrix comparing all dilutions to each other. While the two highest concentrations show a correlation close to the maximum, the similarity starts to decline at higher dilution factors. When the pool is under-sampled (at an average expected copy number of less than 1), the signal changes and eventually becomes artifactual at higher dilutions. These amplification artifacts occur at very high PCR Ct-values and while they can be similar to each other, they no longer bear significant similarity to the correct output, corresponding to what would be considered a failed authentication.

This is further shown in Figure 3b, which plots the correlation relative to the internal reference against the expected copy number in the reaction, showing that at a copy number of less than 1, the similarity declines below the previously observed distribution for same inputs. Combined, these results show the dilution-dependent signal decline and loss of specificity. To achieve a similarity to the reference close to one, i.e., for successful authentication, a copy number above 1 is required, and for optimal results 10–100 copies are desirable. These results also imply that the pool cannot be diluted by a malicious actor, as measuring a lower copy number affects the Ct-value of the PCR step (as shown in Figure S3), and eventually leads to a complete loss of signal specificity of the readout. These factors offer additional protection and security in an anticounterfeit setting.

### Product Labeling Through CUF-Containing Silica Nanoparticles

Based on these results, we then developed a procedure to integrate and extract a CUF into a chemical product for authentication. As an example for a typical orally administered active pharmaceutical ingredient, acetaminophen (more commonly known as paracetamol), was chosen due to its wide application as an analgesic drug and its previous use as a model substance for DNA labeling.^28^ In order to protect and stabilize the DNA against temperature and other environmental influences, as well as potentially harmful further processing steps, the CUF was encapsulated in silica particles before product integration. Amorphous silica is a food additive approved in Europe as E551,^40^ and is also used for oral drug delivery.^41^ Along with the proven protective effect against DNA degradation,^42^ these properties make silica an ideal matrix for the intended application. DNA is equally unproblematic for oral consumption. With the length of the sequences being <100 bp, they are too short to be biologically active and due to the random composition, each sequence is only present at an extremely low copy number. Moreover, the gastrointestinal tract breaks down synthetic DNA identically as any other DNA present in food.^43^

**Figure 4:**
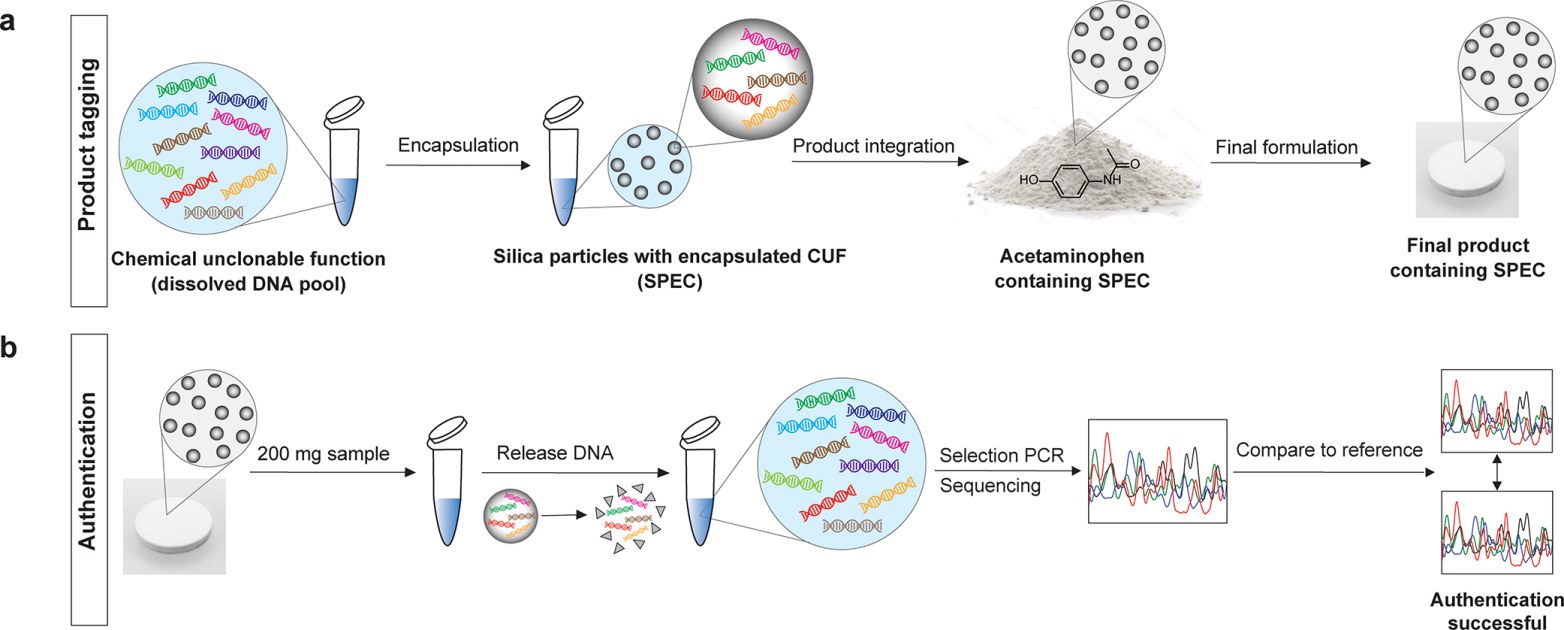
Acetaminophen tagging with silica-encapsulated chemical unclonable functions. (a) Schematic depiction of product integration procedure. (b) Extraction and authentication workflow.

The procedure of labeling a product with a CUF is schematically described in Figure 4a. If a product needs to be authenticated, the CUF particles can be retrieved and the DNA extracted from the surrounding matrix to allow for readout, as shown in Figure 4b.

**Figure 5:**
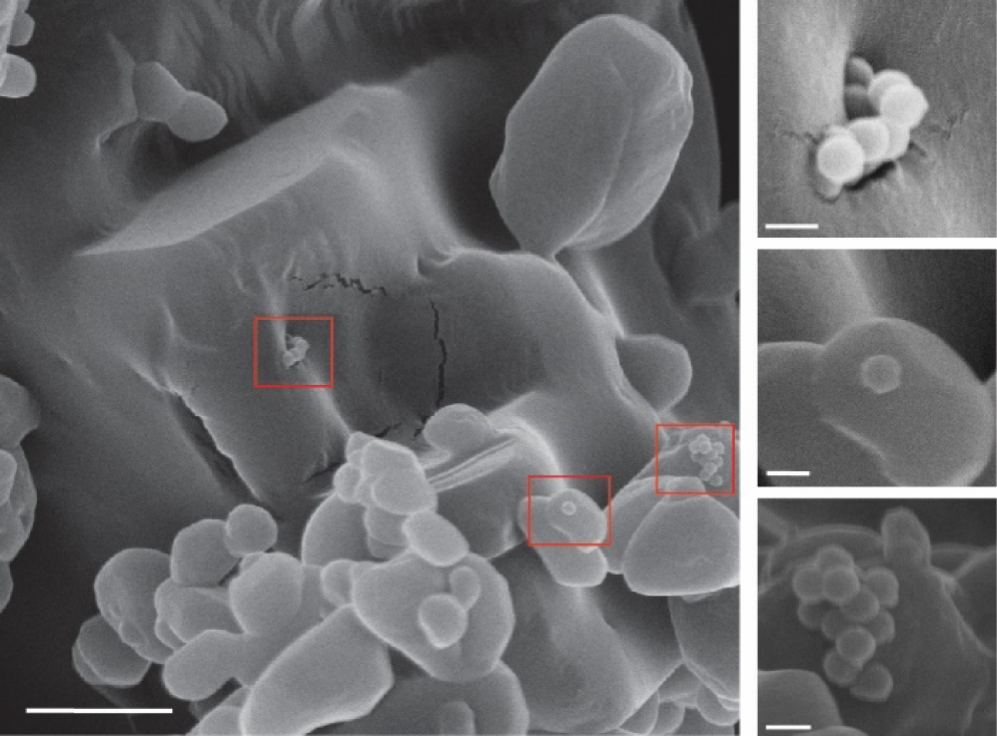
Scanning electron microscopy of silica encapsulated CUF DNA in an acetaminophen formulation. Red squares indicate the image portions that are further magnified on the right. Large scale bar is 2 μm, small scale bars are 200 nm.

The nanoparticles with encapsulated CUF DNA were synthesized using an adapted protocol based on Paunescu et al.,^24^ resulting in monodisperse nanoparticles *approx.* 150 nm in size, with 9.4 ng DNA loaded per μg of silica. The particles were then mechanically mixed into a formulation of acetaminophen at a concentration of *approx.* 5 μg/g (*ca* 5 ppm). Figure 5 shows scanning electron microscopy (SEM) images of the mix. Small clusters of silica particles are visibly embedded in the formulation, sitting on the surface of larger pieces of the drug matrix.

From this mix, several pills were pressed. For authentication, i.e., readout of the enclosed CUF and comparison to a reference, several 200 mg subsamples were taken from each pill and subsequently analyzed.

**Figure 6:**
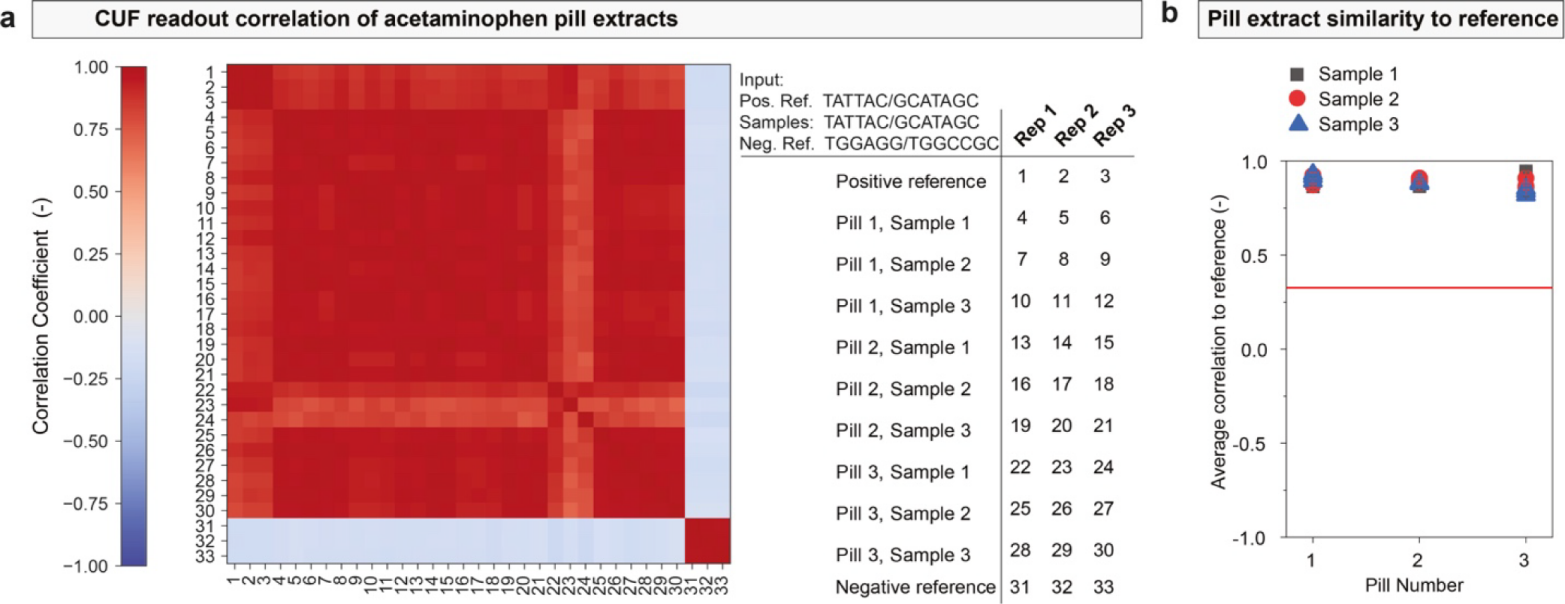
Analysis and stability of product-integrated chemical unclonable functions (CUFs) in an acetaminophen formulation. (a) Matrix comparing correlation between acetaminophen pill extracts and reference samples, as indicated in the table. Three independent readouts were performed per sample and reference (Rep 1, Rep 2, Rep 3). A total of 9 samples stemming from 3 individual pills were taken. The positive reference corresponds to measurement of the same input with pure CUF DNA (i.e., not encapsulated or product-associated), and the negative reference to an analogous measurement using a different, arbitrary input. (b) Outputs measured with extracts from three pills as compared to the positive reference. Each data point refers to the mean correlation of a given readout as compared with the three positive reference samples. The red line indicates the defined threshold above which a product will be considered authentic, based on the previously measured distribution of unlike input correlation.

Workup consisted of isolating the silica nanoparticles and releasing the CUF DNA from the silica matrix. The retrieved DNA was then analyzed by subjecting it to an arbitrarily selected challenge. The responses of different subsamples from several pills were then compared to the reference. For each subsample, three independent CRP measurements were performed. Figure 6a shows the outcome of all analyses in a cross-comparison matrix along with the positive and negative reference measurements. Figure 6b shows the correlation coefficient of each sample in comparison to the positive reference. All pills and their analyzed samples show similar correlations to the reference, with the measured average correlations ranging from 0.82 to 0.95. All measurements are far away from the previously determined correlation range for unlike inputs, whereby the upper bound of the 99% confidence interval (as shown in Figure 2d) was defined as the threshold to assess authenticity.

Overall, these results indicate that the chosen concentration as well as the sampling method are appropriate for the proposed authentication procedure. One advantage of CUFs is that ample copies can be generated before switching the DNA into its unclonable state,^37^ meaning large batches are supported to label product amounts up to the multiton scale. As the subsample size of 200 mg is rather small (corresponding to only a fraction of an average dose) and analyzing one or more entire pills per product identification would be feasible, the lower limit of DNA concentration in a pill could likely be further reduced.

### Stability and Shelf Life Assessment

**Figure 7:**
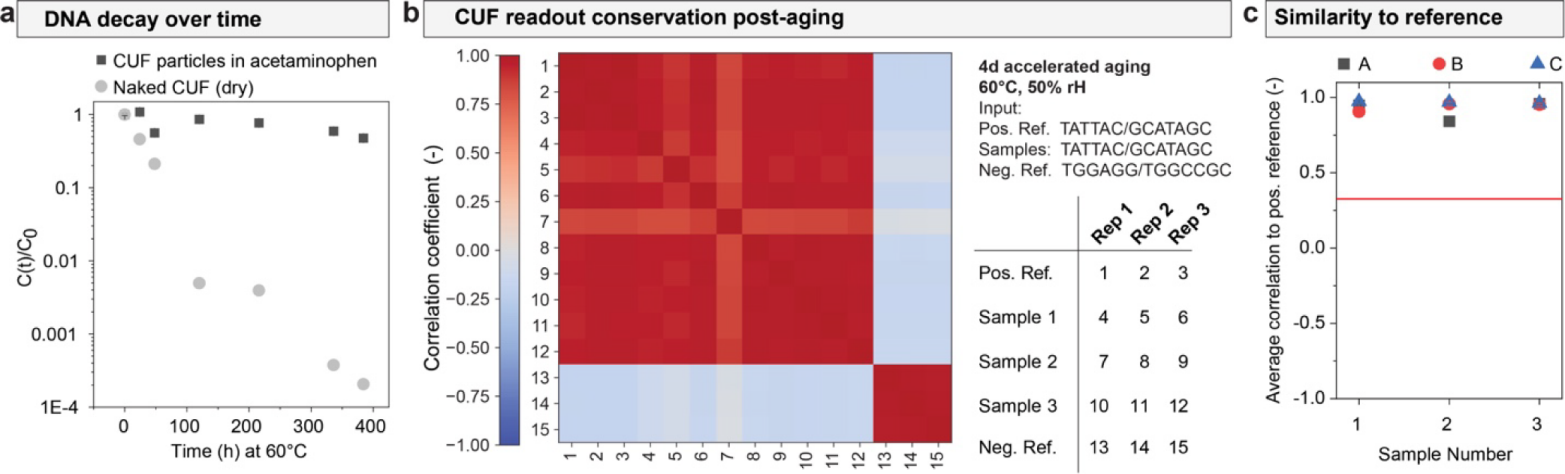
CUF performance after accelerated aging. (a) Accelerated aging data for CUF particles in acetaminophen and naked CUF (dried, nonencapsulated DNA). Relative DNA concentration as measured by qPCR is plotted against the storage time at 60 °C. Data points represent the mean of three measured samples per time point. (b) Matrix showing the correlation between outputs generated with an encapsulated CUF that was mixed into acetaminophen and subjected to accelerated aging. Positive and negative references are displayed for comparison, as indicated in the table. The positive reference corresponds to measurement of the same input with pure CUF DNA (i.e., not encapsulated or product-associated) that has not been subjected to aging and was stored at −20 °C. The negative reference refers to an analogous measurement, but using a different, arbitrary input. (c) Outputs measured with the aged samples after acetaminophen extraction compared to the positive reference. Each data point refers to the mean correlation of a given sample as compared with the three positive reference samples. A, B and C refer to three independently measured outputs. The red line indicates the defined threshold above which a product will be considered authentic, based on the previously measured distribution of unlike output correlation.

In order to assess stability and shelf life of the CUF tags, accelerated aging experiments were performed. First, the general stability of CUF encapsulates was tested, whereby the encapsulate mixed into acetaminophen was compared to dried CUF DNA (without matrix). The samples were stored at 60 °C for up to 16 days, at a constant relative humidity of 50%. After the high-temperature exposure, the overall concentration of remaining DNA was measured by qPCR. Figure 7a shows that the encapsulates mixed into acetaminophen decay several orders of magnitude slower than the dried, naked DNA. Applying the Arrhenius law to the data and assuming an activation energy of 155 kJ/mol,^42^ the calculated shelf life at 25 °C increases 12-fold, from *approx.* 0.36 years for dried DNA to 4.32 years for the encapsulates mixed into acetaminophen. The term shelf life refers to t90, i.e., the time point at which 90% of the DNA is still intact. t90 is also the metric commonly used to determine the expiry date of drug products.^43^ Consequently, the stability of the encapsulated DNA is comparable to the one of the commercial acetaminophen product used in this study, which expires 3 years after the manufacturing date.^44^

To show that the DNA is not only detectable, but still fully functions as a CUF after an extended time period, a sample of the CUF-acetaminophen mixture was stored for 4 days at 60 °C before measuring a challenge-response-pair. This simulates an aging period of *approx.* 8 years at room temperature, which is significantly longer than the shelf life calculated above. As shown in Figure 7b,c, the readout correlations are similar to the results achieved with nonaged CUF DNA extracted from the formulated pill (Figure 6b), and applying the same metric as above, authentication would still be successful well beyond the regulatory shelf life of most drugs.

## Conclusion

In conclusion, our study shows that chemical unclonable functions (CUFs) based on random DNA pools can be used to label and authenticate chemical products. Specifically, we developed a simplified workflow to evaluate product authenticity via Sanger sequencing electropherogram comparison, showing that the introduced method is successful in distinguishing challenge-response-pairs at the maximum resolution. This workflow is faster and more automated than the previously reported method, resulting in an estimated cost of <5 USD per evaluation by Sanger sequencing. Moreover, the employed method of automated peak alignment, Fourier-fitting and correlation analysis also has general implications for automated spectral comparison. Finally, we successfully demonstrated that acetaminophen pills can be labeled homogeneously with silica-encapsulated CUFs, showing that readout is consistent, reliable and stable over time, requiring small amounts of product for authentication with a DNA concentration in the ng/g range. Overall, our results demonstrate the feasibility of using chemical unclonable functions as an anticounterfeit tag of pharmaceutical products. The method has a high level of security against replication and dilution attacks and enables labeling on the product level, the batch level or even the dose level. The implemented formulation is versatile in its use and is suitable for solid, liquid or gel formulations and polymers. Thus, chemical unclonable functions are a promising tool for the fight against counterfeit products in pharmaceuticals and beyond.

## Methods

Library designs and primer sequences are listed in Figure S1 and Tables S1–S3. All DNA was ordered from Microsynth AG (Balgach, Switzerland).

### CUF Synthesis

#### Generation

The DNA pools comprising different CUFs were sourced from a larger random library by sampling the respective sequence number and copying them by PCR. The library was ordered from the supplier in 5 nmol dried aliquots. To generate a CUF, an aliquot was dissolved in Millipore water (type 1, 18.2 MΩ·cm at 24 °C, Milli-Q; Merck, Darmstadt, Germany) to a concentration of 100 μM. Dilution series were performed to achieve the necessary concentration for pipetting the desired number of unique sequences (10^8^ sequences in this study, corresponding to *approx.* 1.66·10^–16^ mol). The PCR mix contained 1.66 μL of a 10^–16^ M solution, 10 μL 1× KAPA SYBR FAST qPCR master mix (KAPA Biosystems, Wilmington, USA) and 1 μL of a 10 μM solution of the forward (fw3) and reverse primer (rx2), respectively (Microsynth AG, Balgach, Switzerland), plus 6.34 μL of PCR-grade water. All dilutions and the final reaction mix were prepared in a laminar flowbench. The PCR program consisted of 95 °C for 180s, followed by cycles of 95 °C for 15s, 30 s annealing at 56 °C and 30 s elongation at 72 °C. The reaction was then purified using the DNA Clean and Concentrator kit (Zymo Research, Irvine, CA, USA).

#### Amplification

To generate the desired amount of a CUF for subsequent labeling experiments, further PCRs were run to amplify the sequences to a higher copy number. One reaction well contained 1 ng of template DNA (the purified CUF from the previous step), 10 μL 1× KAPA SYBR FAST qPCR master mix (KAPA Biosystems, Wilmington, USA) and 1 μL of a 10 μM solution of the forward (fw3) and reverse primer (rx2), respectively (Microsynth AG, Balgach, Switzerland), in a total volume of 20 μL. The PCR program consisted of 95 °C for 180s, followed by cycles of 95 °C for 15s, 30 s annealing at 56 °C and 30 s elongation at 72 °C. The reaction was stopped as soon as the fluorescence signals reached a plateau. The reactions were then purified using the DNA Clean and Concentrator kit (Zymo Research, Irvine, CA, USA). The number of reactions was adapted to the desired batch size.

#### Restriction Digest

After generating the desired amount of copies of a CUF, the outer constant sequences were removed through a restriction digest with the PleI endonuclease. One μg DNA was digested in a reaction mix containing 1× rCutSmart buffer, 50 U PleI enzyme (5U/ μL) in Millipore water at a total volume of 50 μL. The enzyme and buffer were purchased from New England Biolabs (Ipswich, MA, USA). The reaction mix was prepared on ice, then incubated at 37 °C for 70 min. Analytical agarose gel electrophoresis was performed to confirm that no undigested product remained after stopping the reaction, on an E-Gel EX gel (2% agarose) with a Power Snap Electrophoresis Device (Thermo Fisher Scientific, Waltham, MA, USA).

#### Blunting

The PleI-digested DNA was treated with Sequenase (Thermo Fisher Scientific, Waltham, MA, USA) and degenerate 2’3′-dideoxy nucleotides (ddNTP Set, Cytiva, Marlborough, MA, USA) to blunt the 5′-overhang ends. The reaction mix contained 300 ng of purified DNA, 2X reaction buffer, 300 μM ddNTP mix and 13 U of the enzyme, in a total volume of 100 μL. The reaction was performed at 37 °C for 1 min and purified using the DNA Clean and Concentrator kit (Zymo Research, Irvine, CA, USA).

### CUF Operation

The PCR selection step was performed in a reaction mix containing 10 μL KAPA SYBR FAST qPCR master mix (KAPA Biosystems, Wilmington, USA), 1 μL of each input primer (forward and reverse, 10 μM), 1 μL CUF DNA (1 ng/ μL) and 7 μL Millipore water. For thermal cycling, a touchdown sequence consisting of 15 s denaturing at 95 °C, annealing at 38 to 48 °C for 30 s and elongation at 72 °C for 30 s. The annealing temperature started at 48 °C and was reduced by 1 °C for each consecutive cycle until reaching 38 °C in the 11th cycle. 34 more cycles were performed at this constant annealing temperature, reaching a total of 45 thermal cycles. After the final cycle, a final elongation step at 72 °C for 120 s was performed.

### Sanger Sequencing Preparation

The purified product from the selection step was diluted to a concentration of 0.1–1 ng/ μL, and then amplified in a preparative PCR to add Sanger adapters for sequencing. The reaction mix comprised a total volume of 20 μL and contained 1 μL DNA stock, 10 μL KAPA SYBR FAST qPCR master mix (KAPA Biosystems, Wilmington, USA) 1 μL of each adapter primer (forward and reverse, 10 μM), and 7 μL Millipore water. The PCR product was purified (refer to section “PCR purification”) and diluted to a concentration of *approx.* 2 ng/ μL, of which a 12 μL aliquot was sent to the service provider for Sanger sequencing (Microsynth AG, Balgach, Switzerland).

### PCR Purification

Purification of preparative PCR steps was performed with the DNA Clean and Concentrator kit (Zymo Research, Irvine, CA, USA). The purified DNA was eluted with Millipore water. Concentration measurements were conducted on a Qubit system (Thermo Fisher Scientific, Waltham, MA, USA). Thermal cycling comprised 11–14 cycles of 95 °C for 180s, followed by cycles of 95 °C for 15s, 30 s annealing at 56 °C and 30 s elongation at 72 °C. The reaction was stopped after the fluorescence signal reached a plateau.

### Data Analysis

Sequencing data were analyzed as .ab1 files using a custom python script. The electropherogram traces were aligned using the constant primer segments, which allow to define the starting and end point of the relevant trace segment corresponding to the output of the chemical function. The constant peaks at either end of the output signal were identified using the “find_peaks” command from the scipy library, then set as the start and end point of the analyzed region, respectively. The output traces were then Fourier-fitted and cross-compared in terms of their correlation coefficient. The overall correlation was defined as the mean of the individual correlations calculated for the four traces (for A, C, G and T).

### Particle Synthesis and Characterization

Two batches, CUF_P1 and CUF_P2, of silica-encapsulated CUF were synthesized, slightly differing in their DNA composition (refer to Table S3) but following the same synthesis procedure. CUF_P2 was used for accelerated aging experiments, CUF_P1 for all other experiments involving particles. CUF DNA was encapsulated in submicron silica particles in an adapted procedure based on Paunescu et al.^24^ SiO_2_ particles (142 ± 4 nm, Lot SiO2-R-L3205–23/1, Microparticles GmbH, Berlin, Germany) were functionalized with N-trimethoxysilylpropyl-N,N,*N*- trimethylammonium chloride (TMAPS) (50 wt % in methanol; abcr). Ten μL of TMAPS were added to 1 mL of a 50 mg/mL particle suspension. The mix was then stirred overnight at room temperature and 900 rotations per minute (rpm). For DNA binding, 16 μL of a 10 μg/ μL TMAPS-functionalized particle suspension were mixed with 75 μL of a 20 ng/ μL CUF DNA solution and 909 μL Millipore water. After vortexing, 0.5 μL N-trimethoxysilylpropyl-N,N,*N*- trimethylammonium chloride (TMAPS) (50 wt % in methanol; abcr) and 0.5 μL tetraethyl orthosilicate (TEOS) (≥99.0%; Sigma-Aldrich) were added to the mix, followed by 4h agitation at room temperature, 900 rpm. Another 4 μL of TEOS (≥99.0%; Sigma-Aldrich) were added to the reaction, which was then again agitated at 900 rpm for 6 dayss

### Production and Analysis of Pills

CUF-containing acetaminophen pills were generated from a commercially available acetaminophen product (Dafalgan 500 mg effervescent tablets, UPSA Switzerland AG, Zug, Switzerland). 3.26 g of the formulated drug product was ground to a fine powder in a mortar. 150 μL of a 100 ng/μL suspension of particle batch CUF_P1 (synthesis described above) in 2-propanol (≥99.8% (GC), ACS reagent, Sigma-Aldrich) were pipetted onto the powder in 15 × 10 μL portions. The powder was then mechanically mixed/ground to achieve a homogeneous distribution and association of the particles with the product. From this mix, 3 × 1 g pills were pressed, simulating the manufacture of individual doses from an initial formulation. For authentication, each pill was again destroyed and ground to a fine powder. Three × 200 mg subsamples were taken from each pill. The product was then slowly dissolved in a mix of 750 μL Millipore water and 500 μL EtOH (absolute for analysis EMSURE ACS, ISO, Reag. Ph Eur, Merck KGaA, Darmstadt, Germany) in a 1.5 mL Eppendorff tube. After bubbling had ceased, the mix was centrifuged for 5 min at 15 000 rpm, after which the supernatant was discarded without disturbing the pellet. One mL Millipore water was added to the pellet, followed by short vortexing and centrifugation at 15 000 rpm for 5 min. The supernatant was again discarded carefully. To the barely visible traces of a pellet, 4 μL of buffered oxide etch (0.03 wt % ammonium hydrogen difluoride (NH_4_FHF, pure; Merck) and 0.02 wt % ammonium fluoride (NH_4_F, puriss.; Sigma-Aldrich)) followed by 16 μL Millipore water were added. After vortexing, the solution was diluted with another 80 μL H_2_O, then placed in an ultrasonic bath for 10 min. For analysis, PCR using a pair of input primers was performed in analogy to the previously described procedure (refer to “CUF operation”). Per 200 mg pill extract, three evaluations were performed. Each reaction mix consisted of 10 μL KAPA SYBR FAST qPCR master mix (KAPA Biosystems, Wilmington, USA), 1 μL of each input primer (forward and reverse, 10 μM), 5 μL sample solution and 3 μL Millipore water.

### Accelerated Aging

For aging of pure DNA, twenty-one glass vials were filled with 2 μL of a 0.75 ng/ μL CUF-DNA. The vials were vacuum centrifuged at 45 °C for 1 h to dry the DNA. For aging of encapsulated CUF, 60 μL of a 100 ng/μL particle suspension (CUF_P2, synthesis described above) were mixed into 1.2 g acetaminophen (98.0–102%, USP grade, Sigma-Aldrich, Burlington, Massachusetts, United States) for 5 min by using a pestle and mortar. Twenty-one vials were filled with 40 mg (±5%) of the acetaminophen mix. Three vials of each set were stored at 4 °C as controls. The 2 × 18 remaining vials were stored in a desiccator containing a reservoir of a saturated NaBr solution to maintain 50% relative humidity. The desiccator was placed in an oven at 60 °C. At each of the six predefined time points, three vials of each set were transferred to 4 °C. For analysis, to each vial containing pure DNA, 98 μL of mQ water were added, followed by 10 min of sonication. The entire volume of each vial was then transferred to 1.5 mL Eppendorf tubes, to which 2 μL of buffered oxide etch (0.03 wt % ammonium hydrogen difluoride (NH_4_FHF, pure; Merck) and 0.02 wt % ammonium fluoride (NH_4_F, puriss.; Sigma-Aldrich)) were added. The tubes were subsequently vortexed and sonicated for 10 min prior to analysis. For the acetaminophen-containing vials, the powder was first dissolved in 500 μL EtOH (absolute for analysis EMSURE ACS, ISO, Reag. Ph Eur, Merck KGaA, Darmstadt, Germany), followed by 10 min of sonication, then the entire volume was transferred to 1.5 mL Eppendorf tubes. The tubes were centrifuged for 5 min at 15 000 rpm. The supernatant containing the dissolved acetaminophen was discarded, and 98 μL of Millipore water and 2 μL of buffered oxide etch (0.03 wt % ammonium hydrogen difluoride (NH_4_FHF, pure; Merck) and 0.02 wt % ammonium fluoride (NH_4_F, puriss.; Sigma-Aldrich)) were added to the pellet, followed by vortexing and 10 min of sonication. All samples were then analyzed using qPCR. The reaction mix contained 10 μL 2× KAPA SYBR FAST master mix (KAPA Biosystems, Wilmington, USA), 1 μL each of the two primer solutions (10 μM, 0F and rv2), 3 μL Millipore water and 5 μL of the respective sample solution. Technical triplicates were measured of each sample. The thermal cycling program consisted of 3 min preincubation at 95 °C, followed by 45 cycles of 15 s melting at 95 °C, 30 s annealing at 56 °C, and 30s of elongation at 72 °C.

### Accelerated Aging with Subsequent CUF Readout

For aging of encapsulated CUF, 20 μL of a 1000 ng/μL particle suspension (CUF_P2, synthesis described above) were mixed into 800 mg acetaminophen (98.0–102%, USP grade, Sigma-Aldrich) for 5 min by using a pestle and mortar. Three 1.5 mL Eppendorf tubes were filled with 40 mg (±5%) of the acetaminophen mix each. The tubes were stored with open lids in a desiccator containing a reservoir of a saturated NaBr solution to maintain 50% relative humidity. The desiccator was placed in an oven at 60 °C.

For workup, the powder was first dissolved in 500 μL EtOH (absolute for analysis EMSURE ACS, ISO, Reag. Ph Eur, Merck KGaA, Darmstadt, Germany) under vortexing. The tubes were then centrifuged for 5 min at 15 000 rpm. The supernatant containing the dissolved acetaminophen was discarded, 4 μL of buffered oxide etch (0.03 wt % ammonium hydrogen difluoride (NH_4_FHF, pure; Merck) and 0.02 wt % ammonium fluoride (NH_4_F, puriss.; Sigma-Aldrich)) followed by 16 μL of Millipore water were added to the pellet, followed by vortexing and 10 min of sonication. Finally, another 80 μL of water were added.

For analysis, PCR using a pair of input primers was performed in analogy to the previously described procedure (refer to “CUF operation”). Per 40 mg aged acetaminophen sample, three evaluations were performed (the equivalent of 9 readouts in total). Each reaction mix consisted of 10 μL KAPA SYBR FAST qPCR master mix (KAPA Biosystems, Wilmington, USA), 1 μL of each input primer (forward and reverse, 10 μM), 5 μL sample solution and 3 μL Millipore water.

## Supporting Information Available

Figures S1–S3, describing sequence design, data processing and Ct-dependency of pool copy numbers; Tables S1–S3, listing used DNA sequences, primers and particle batches; Supporting Note S1 on the comparison of different pharmaceutical identifiers and anticounterfeit methods (PDF)

## Abbreviations

PCR: polymerase chain reaction
CRP: challenge-response-pair
CUF: chemical unclonable function
PUF: physical unclonable function
NGS: next generation sequencing
SEM: scanning electron microscopy
