# Supplementary material for: DNA-Based Chemical Unclonable Functions for Cryptographic Anticounterfeit Tagging of Pharmaceuticals
Source: ACS Nano. 2024 Oct 22;18(44):30774–85. doi: 10.1021/acsnano.4c10870 (PMC11544705; doi:10.1021/acsnano.4c10870)
Supplement: Supplementary file 1 — nn4c10870_si_001.pdf [file nn4c10870_si_001.pdf]

Supporting Information

DNA-based Chemical Unclonable Functions for cryptographic anti-counterfeit tagging of pharmaceuticals

Anne M. Luescher, Wendelin J. Stark, Robert N. Grass*

*Corresponding author

robert.grass@chem.ethz.ch

Institute of Chemical and Bioengineering, ETH Zurich, Vladimir-Prelog-Weg 1, 8093 Zurich, Switzerland

Table of contents

Figure S1. Chemical unclonable function (CUF) design showing the different processing stages from CUF generation to CUF operation.

Figure S2. Data processing example of Sanger electropherograms.

Figure S3. Ct value difference measured in selection PCR with a constant input (GTCTCA/CCGGTTG) and different copy numbers of the CUF pool present in the reaction.

Table S1. List of general primers

Table S2. List of input primers

Table S3. CUF particle batches

Supporting Note S1: Comparison of different pharmaceutical identifiers and anti-counterfeit methods

1. Experimental section

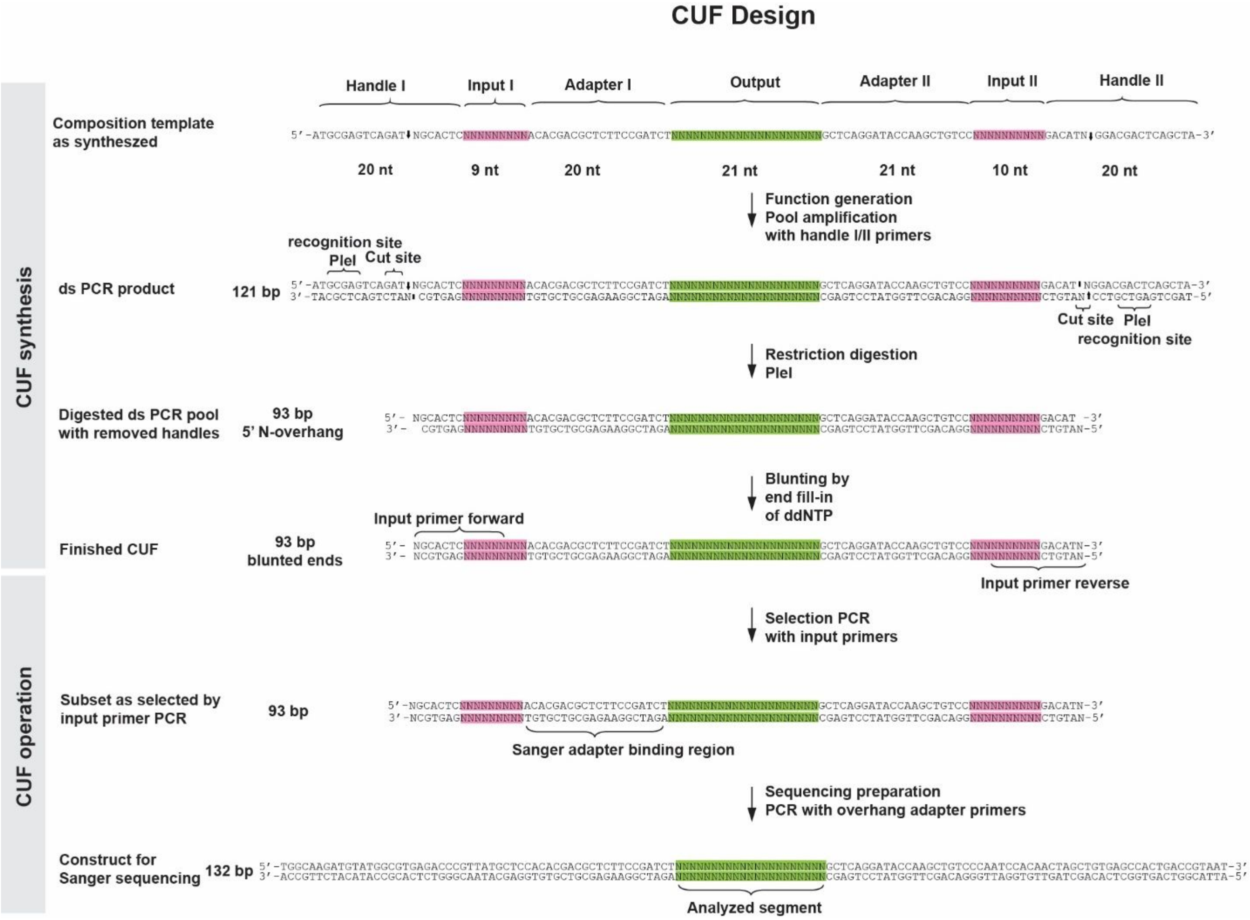

Figure S4. Chemical unclonable function (CUF) design showing the different processing stages from CUF generation to CUF operation. The library composition is ordered from an external provider as a single strand construct. The library contains handle sequences at both ends, allowing for global amplification of all individual sequences. Each strand contains three random portions, two of which are labelled as input regions, the third one, situated at the center of the sequence, as the output. The inputs are separated from the output by two adapter sequences. To use such a library as a chemical unclonable function, the sequences are first PCR-amplified to get multiple double-stranded copies of the initially single-stranded and unique oligos. To prevent further copying, the sequences are then truncated through a restriction digest and blunted with dideoxy-NTPs. This truncated product can then be distributed and used as the CUF. For operation, two input primers are added to a PCR reaction and partially bind to the input sections of the small subset of sequences within the entire CUF pool that are Watson-Crick complementary to the primers. The

thus selected sequences are exponentially amplified, before adding adapter sequences via a second PCR using overhang primers. The final construct – now only containing the adapter sequences and the output – can then be Sanger sequenced.

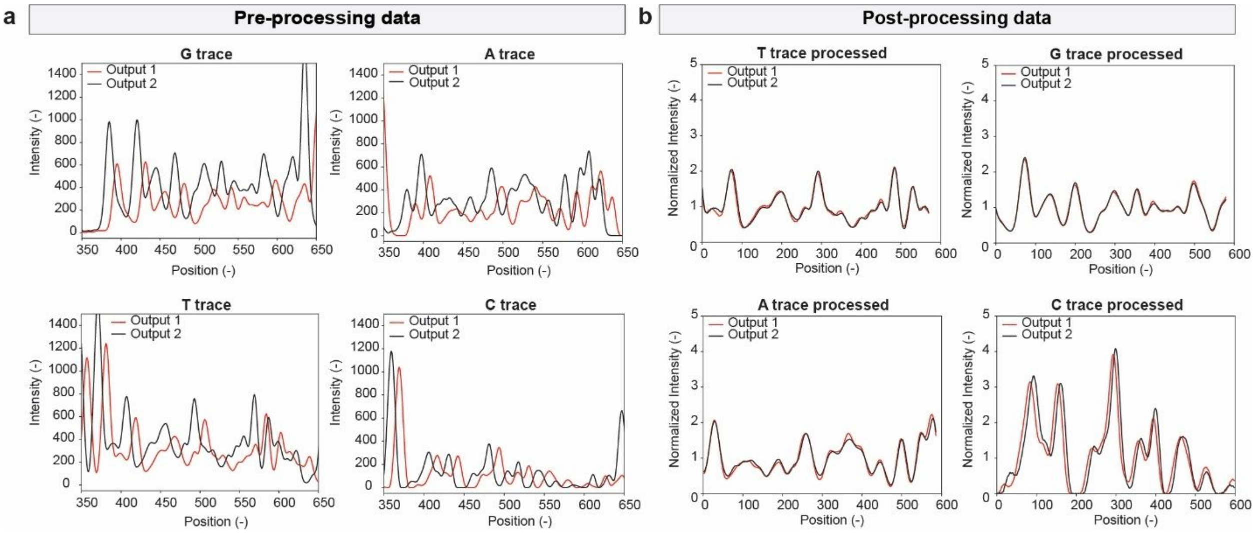

Figure S5. Data processing example of Sanger electropherograms. (a) Unprocessed raw data of the four traces (representing G, A, T and C) of two separately measured outputs, both generated with CUF 1 and inputs GACTTC/GCAAAGG. (b) Traces after processing using peak identification, normalization and re-sampling.

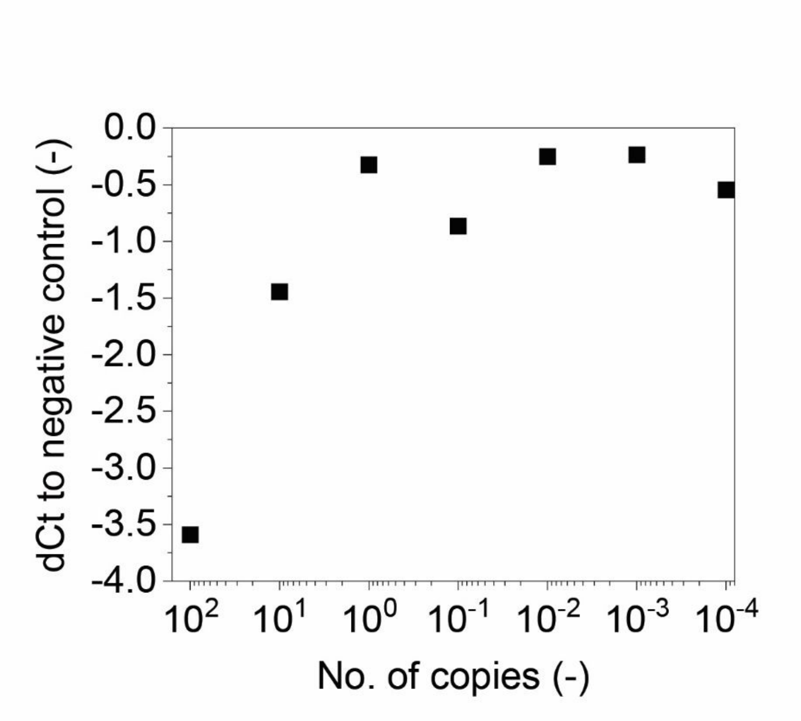

Figure S6. Ct value difference measured in selection PCR with a constant input (GTCTCA/CCGGTTG) and different copy numbers of the CUF pool present in the reaction. The y-axis shows the difference between the average Ct value of two repetitions and the average Ct value of the respective negative controls (PCR reaction mix without added DNA).

Table S4 List of general primers

Primer designation	Primer sequence (5'-3')	Primer type
fw3	ATGCGAGTCAGATNGCACTC	Handle I
rx2	GACATNGGACGACTCAGCTA	Handle II
0F	ACACGACGCTCTTCCGATCT	Adapter I
rv2	GGACAGCTTGGTATCCTGAGC	Adapter II
Sanger adapter fw	TGGCAAGATGTATGGCGTGAGACCCGTTATGCTCC ACACGACGCTCTTCCGATCT green = overlap with Adapter I	Sanger adapter I
Sanger adapter rv	ATTACGGTCAGTGGCTCACAGCTAGTTGTGGATTG GGACAGCTTGGTATCCTGAGC blue = overlap with Adapter II	Sanger adapter II
Sanger sequencing primer	TGGCAAGATGTATGGCGTGAG	Sanger sequencing primer

Table S5 List of input primers

Primer designation	Primer sequence (5'-3')	Primer type
Lib5_infw1	NGCACTCCGATTA	forward
Lib5_inrv1	NATGTCTTCGGAC	reverse
Lib5_infw2	NGCACTCGCTTAC	forward
Lib5_inrv2	NATGTCGAGCGGC	reverse
Lib5_infw3	NGCACTCTCTAAC	forward
Lib5_inrv3	NATGTCCCGGTTG	reverse
Lib5_infw4	NGCACTCTGACAA	forward
Lib5_inrv4	NATGTCTGGTCGA	reverse
Lib5_infw5	NGCACTCCGTCTG	forward
Lib5_inrv5	NATGTCACGGAGA	reverse
Lib5_infw6	NGCACTCTATTAC	forward
Lib5_inrv6	NATGTCTGAGTCA	reverse
Lib5_infw7	NGCACTCTAGTCA	forward
Lib5_inrv7	NATGTCCGCGGTC	reverse
Lib5_infw8	NGCACTCGTCTCA	forward
Lib5_inrv8	NATGTCCGTCTCA	reverse
Lib5_infw9	NGCACTCTGGAGG	forward
Lib5_inrv9	NATGTCCATAGC	reverse
Lib5_infw10	NGCACTCCACCGGC	forward
Lib5_inrv10	NATGTCGGCCGC	reverse
Lib5_infw11	NGCACTCTATTTC	forward
Lib5_inrv11	NATGTCTTAGGGA	reverse
Lib5_infw12	NGCACTCTACGGA	forward
Lib5_inrv12	NATGTCCGTTGGG	reverse
Lib5_infw13	NGCACTCGACTTC	forward
Lib5_inrv13	NATGTCGCAAAGG	reverse
Lib5_infw1.1	NGCACTCTGATTA	forward
Lib5_inrv1.1	NATGTCCTCGGAC	reverse

Segments marked in red indicate input bases

Table S6 CUF particle batches

Primer designation	Encapsulated construct (dsDNA)
CUF_P1	5’-NGCACTCNNNNNNNNNACACGACGCTCTTCCGATCTNNNN NNNNNNNNNNNNNNNNNGCTCAGGATACCAAGCT GTCCNNN NNNNNNNGACATN-3’<br>3’-NCGTGAGNNNNNNNNNTGTGCTGCGAGAAGGCTAGANNNN NNNNNNNNNNNNNNNNNCGAGTCCTATGGTTCGACAGGNNNN NNNNNNCTGTAN-5’
CUF_P2	5’TGCGAGTCAGATNGCACTCNNNNNNNNNACACGACGCTCTTCC GATCTNNNNNNNNNNNNNNNNNNNNNGCTCAGGATACCAAGCT GTCCNNNNNNNNNNGACATNGGACGACTCAGCTA-3’<br>3’TACGCTCAGTCTANCGTGAGNNNNNNNNNTGTGCTGCGAGAAGG CTAGANNNNNNNNNNNNNNNNNNNNNCGAGTCCTATGGTTCGA CAGGNNNNNNNNNNCTGTANCCTGCTGAGTCGAT-5’

Supporting Note S1: Comparison of different pharmaceutical identifiers and anti-counterfeit methods

RFID: Bansal *et al.*[1] mention RFID chips as a common anti-counterfeit technology used in pharmaceutical industry, whereby the tag is *e.g.* associated to cases or pallets. They state that RFIDs are difficult to duplicate or alter, and that they possess a high data storage capacity. In a technical overview of RFID, Weinstein[2] more specifically benchmarks the typical information content of a tag at 2 kbit. This is categorized as "too small to hold much more complex information than identification and history information". However, in the context of anti-counterfeit tags we still consider this as a high information content, since among the compared technologies 2 kbits still rank relatively high, as also stated by Bansal *et al.*[1] The readout works via electromagnetic signals[2], therefore the time to complete an authentication using a scanner is as low as seconds. In terms of security, Li *et al.*[3] state that the information contained in RFID tags can be encrypted, but is vulnerable to common malicious attacks.

Nanomaterials: This is a relatively broad category and we focus on the use of screen-printed nanoparticles and fluorescent 3D QR codes based on nanoparticles in this evaluation, since they have been explicitly applied to pharmaceutical products. Campos-Cuerva *et al.*[4] use screen-printed nanoparticles as tags, which can be read using optical and magnetic signals, enabling quick readout. They state that properties of a combination of nanoparticles are difficult to replicate. You *et al.*[5] use fluorescent nanoparticle printing for the generation of 3D QR codes (using 3 fluorescence channels), which can be read with a smartphone app with near-immediate readout.

Both technologies do not intrinsically encrypt the information stored in the tag. Neither of the publications directly mentions the theoretical or practical information storage capacity. However, Sharief *et al.*[6] provided a comparison of physical anti-counterfeit technologies similar to this classification and marks the storage density for nanoparticle-based technologies generally as low. Moreover, we can infer at least the practical level of information content from the presented experimental use cases. Campos-Cuerva *et al.* printed a 14 letter word with different combinations of nanoparticles, using a combination of 3 types yielding different spectra. As there is a limited number of possible combinations and a limited print capacity, we estimate the directly accessible information content to be in the range of bytes to kilobytes. You *et al.* stored a weblink, a low-resolution picture and a short text, which would range in the order of bytes to kilobytes. This also aligns with the reported storage capacity of QR codes, which is in the kilobyte range.[7]

Edible PUF: Leem *et al.*[8] report the generation of edible physical unclonable functions based on fluorescent silk proteins film-printed on capsules. These prints can be analyzed by optical scanning. While the authors do not specify readout time, the optical nature of the analysis suggests a readout within seconds, provided suitable fluorescence detectors and an integrated software for automated image analysis are available. The method is based on random deposition of the fluorescent silk microparticles and is as such unclonable. As is typical for physical unclonable functions, cryptographic keys are extracted from this random information, leading to a very high level of security. Each key has a length of 256 bits in their implementation, but the theoretical encoding capacity is calculated to be 2^{120} bits, which is extremely high.

DNA barcode: DNA barcodes are relatively short DNA fragments that are admixed or otherwise attached to products as identifiers. To authenticate the product, a PCR reaction, or alternatively DNA sequencing, is performed that detects the respective fragment. Altamimi *et al.*[9] and Jung *et al.*[10] use single fragments of DNA of up to 200 bp, while Liu *et al.*[11] uses a 720 bp fragment. Calculating with an information density of 2 bits/base, this corresponds to an information content of <200 bytes, and thus a very low encoding capacity. Moreover, none of the reported methods are encrypted, and the effort to copy the label only comprises a single sequencing and synthesis reaction, which is therefore only a moderate effort compared to duplication of RFIDs, nanomaterial signatures or even unclonable functions.

DNA unclonable functions (this work): We use DNA-based unclonable functions, which are generated based on a random physical process and can therefore not be cloned deterministically and as such provide a very high level of information security. Readout uses PCR and sequencing technologies, providing an evaluation within hours. We demonstrate a protocol to stably integrate the tag into the drug product. In contrast to DNA barcodes, the tag consists of 100 million DNA sequences comprising 40 random base pairs each, providing an encoding capacity of a gigabyte (100 million x 40 base pairs x 2 bits/base pair).

References

(1) Bansal, D.; Malla, S.; Gudala, K.; Tiwari, P. Anti-Counterfeit Technologies: A Pharmaceutical Industry Perspective. *Sci. Pharm.* **2013**, *81,* 1-14.
(2) Weinstein, R. RFID: A Technical Overview and its Application to the Enterprise. *IT Prof.* **2005**, *7,* 27-33.
(3) Li, H.; Chen, Y.; He, Z. The Survey of RFID Attacks and Defenses. *2012 8th International Conference on Wireless Communications, Networking and Mobile Computing* **2012**, 1-4.
(4) Campos-Cuerva, C.; Zieba, M.; Sebastian, V.; Martinez, G.; Sese, J.; Irusta, S.; Contamina, V.; Arruebo, M.; Santamaria, J. Screen-Printed Nanoparticles As Anti-Counterfeiting Tags. *Nanotechnology* **2016**, *27,* 095702.
(5) You, M.; Lin, M.; Wang, S.; Wang, X.; Zhang, G.; Hong, Y.; Dong, Y.; Jin, G.; Xu, F. Three-Dimensional Quick Response Code Based on Inkjet Printing of Upconversion Fluorescent Nanoparticles for Drug Anti-Counterfeiting. *Nanoscale* **2016**, *8,* 10096-10104.
(6) Sharief, S. A.; Chahal, P.; Alocilja, E. Application of DNA Sequences in Anti-Counterfeiting: Current Progress and Challenges. *Int. J. Pharm.* **2021**, *602,* 120580.
(7) Tiwari, S. An Introduction to QR Code Technology. In *2016 International Conference on Information Technology (ICIT)*, 2016; pp 39-44.
(8) Leem, J. W.; Kim, M. S.; Choi, S. H.; Kim, S.-R.; Kim, S.-W.; Song, Y. M.; Young, R. J.; Kim, Y. L. Edible Unclonable Functions. *Nat. Commun.* **2020**, *11,* 328.
(9) Altamimi, M. J.; Greenwood, J. C.; Wolff, K.; Hogan, M. E.; Lakhani, A.; Martin, G. P.; Royall, P. G. Anti-Counterfeiting DNA Molecular Tagging of Pharmaceutical Excipients: An Evaluation of Lactose Containing Tablets. *Int. J. Pharm.* **2019**, *571,* 118656.
(10) Jung, L.; Hogan, M. E.; Sun, Y.; Liang, B. M.; Hayward, J. A. Rapid authentication of pharmaceuticals via DNA tagging and field detection. *PLoS One* **2019**, *14,* e0218314.
(11) Liu, Y.; Zhang, C.; Li, X.; Wu, D. A Self-Monitored Fluorescence DNA Anti-Counterfeiting System Based on Silica Coated SYBR Green I/DNA Gelatin Nanoparticles. *J. Mater. Chem. C* **2017**, *5,* 5939-5948.
